# Standardised Tool for the Assessment of Bruxism: Translation, Cultural Adaptation and Pilot Testing in Italy

**DOI:** 10.1111/joor.13882

**Published:** 2024-10-20

**Authors:** Anna Colonna, Frank Lobbezoo, Jari Ahlberg, Alessandro Bracci, Matteo Pollis, Matteo Val, Laura Nykänen, Daniele Manfredini

**Affiliations:** ^1^ School of Dentistry, Department of Medical Biotechnologies University of Siena Siena Italy; ^2^ Department of Orofacial Pain and Dysfunction, Academic Centre for Dentistry Amsterdam (ACTA) University of Amsterdam and Vrije Universiteit Amsterdam Amsterdam The Netherlands; ^3^ Department of Oral and Maxillofacial, Diseases University of Helsinki, Helsinki University Central Hospital Helsinki Finland; ^4^ Department of Neurosciences, School of Dentistry University of Padova Padova Italy

**Keywords:** assessment, awake bruxism, bruxism, bruxism evaluation, sleep bruxism, STAB

## Abstract

**Background:**

Recently, the Standardised Tool for the Assessment of Bruxism (STAB) has been developed for use in clinical and research settings.

**Objectives:**

The aim of the present study is to describe the process of forward and back translation and pilot testing of the STAB into Italian.

**Methods:**

The English version of the STAB was adopted as a template for translation into other languages, according to a step‐by‐step procedure led by the expert STAB bruxism panel and mother tongue experts in the field. In detail, the translation team was made up of 12 subjects: three study coordinators, two forward translators, two back‐translators and five expert panellists.

Following the translation process, a pilot test in patients, dentists and dental students was performed using the ‘probing’ method (i.e., subjects were questioned by the examiners about the perceived content and interpretation of the items) with the aim to assess the comprehensibility of the questions and response options, and the feasibility of the tool.

**Results:**

This paper describes the translation process of the STAB and provides the outcomes of the pilot testing phase and the face validity assessment. The preliminary results suggest that, from a global point of view, the STAB reflects the characteristics required in clinical and research settings.

**Conclusion:**

Thanks to the translation process, the Italian version of the STAB can be assessed on‐field and introduced in the clinical and research field to get deeper into the study of bruxism epidemiology in Italy.

## Graphical abstract

Recently, the Standardized Tool for the Assessment of Bruxism (STAB) has been developed for use in clinical and research settings. Based on the outcomes of the pilot testing as well as of the face validity assessment, the Italian version of the STAB can be introduced in the clinical and research settings to get deeper into the study of bruxism epidemiology in Italy.

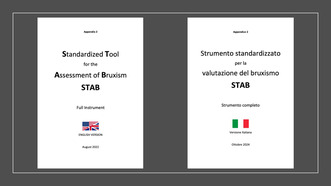

**Abbreviations**
AB: awake bruxism
PSG: polysomnography
SB: sleep bruxism
STAB: Standardised Tool for the Assessment of Bruxism

## 1 Introduction

Several definitions and evaluation protocols are available for bruxism, which may lead to confusion in both the clinical and research settings. Within the past decade, however, a widely accepted consensus has been reached [1, 2, 3, 4, 5]. As a result of the international consensus meetings, the construct of bruxism has been broadened to encompass a wider spectrum of jaw‐muscle activities, not limited to the act of grinding teeth while asleep, and also including longer lasting, prolonged activities that are potentially typical of muscle bracing and teeth clenching [1, 2, 3, 4, 5, 6]. Two separate definitions were suggested according to the circadian rhythm:

- ‘Sleep bruxism (SB) is a masticatory muscle activity during sleep that is characterised as rhythmic (phasic) or non‐rhythmic (tonic) and is not a movement disorder or a sleep disorder in otherwise healthy individuals’.
- ‘Awake bruxism (AB) is a masticatory muscle activity during wakefulness that is characterised by repetitive or sustained tooth contact and/or by bracing or thrusting of the mandible and is not a movement disorder in otherwise healthy individuals’ [1].

In view of this, the strategies adopted up to now for the evaluation of sleep and awake bruxism were re‐evaluated. In particular, in the case of SB, the commonly used polysomnography (PSG) criteria provide only a partial picture of the complex range of jaw‐muscle activities, while for AB, no criteria were available [1, 2, 3, 4, 5, 6, 7, 8, 9, 10].

Based on these premises, the efforts of international experts led to the creation of the Standardised Tool for the Assessment of Bruxism (STAB), an instrument that was developed to provide a multidimensional evaluation of several issues concerning bruxism status, comorbid conditions, aetiology and clinical consequences [8, 11]. In detail, the STAB includes 14 domains, accounting for a total of 66 items and is composed of two main axes split into multiple sections: The Axis A deals with the assessment of bruxism (subject‐based assessment—self‐report, clinically based assessment [signs/symptoms/consequences]—examiner report, instrumentally based assessment—technology report) as well as the potential clinical consequences; the Axis B deals with the aetiological, risk and associated factors and comorbid conditions [8, 11]. Depending on the specific research and/or clinical needs of the operators, some sections of the STAB might specifically be selected. The list of full instruments that is preferred for specific applications is provided in a ToolKit, where also additional tools for possible use can be found [11].

The STAB as well as the original instruments included in the Toolkit were created in the English language, so a structured procedure for translation and cultural adaptation in secondary languages has been developed [12]. Indeed, the sociodemographic and cultural differences may limit the validity with respect to the original English version, because in the absence of a cultural contextualisation, the translation process itself does not guarantee an equivalent validity [13].

Within these premises, the twofold aim of the present paper is: (1) to describe the procedure adopted to translate the STAB into Italian according to the above guidelines and (2) present for the first time some pilot data on the comprehensibility and feasibility of the STAB.

## 2 Methods

### 2.1 Translation Process

Guidelines for the multi‐language translation of the English version of the STAB were employed as a template according to a step‐by‐step procedure defined and led by the STAB developers and mother‐tongue experts in the field [12]. The translation team included 12 professionals: three study coordinators, two forward translators, two back‐translators and five expert panellists; the role(s) and qualifications are shown in Table 1. For any further details on the strategies needed to select the translation crew (member, criteria, roles, steps), readers are referred to the original guidelines [12].

**TABLE 1:** Overview of the translation crew: Members, type of member, role(s) and qualifications.

Members	Type of member	Role (s)	Qualifications
Daniele Manfredini	Project leader	Review and approval of translation teams Review and approval of translation logs	Dentist Specialty in Orthodontics Bruxism and Temporomandibular Disorders expert MSc in Occlusion and Temporomandibular Disorders PhD in Dental Research Diplomate American Board of Orofacial Pain Co‐Chairman of the international bruxism consensus panel within the International Association for Dental research (IADR)
Anna Colonna	Team leader Study coordinator 2	Composition of translation team Overall responsibility for the entire project Synthesis of the two back‐translations, together with both back‐translators	Dentist Specialty in Orthodontics Bruxism, Sleep‐Related Breathing Disorders and Temporomandibular Disorders expert
Alessandro Bracci	Study coordinator 1	Synthesis of the two forward translations, together with both forward translators	Dentist Bruxism and Temporomandibular Disorders expert Member of the international bruxism consensus panel within the International Association for Dental research (IADR)
Luca Guarda‐Nardini	Study coordinator 3	Identify discrepancies between the common back‐translation and the source document	Medical Doctor Specialty in maxillofacial surgery Specialty in otorhinolaryngology Specialty in dentistry Bruxism and Temporomandibular Disorders expert
Matteo Val	Forward translator 1	Translate the source document into the target language	Dentist Specialty in oral surgery Bruxism and Temporomandibular Disorders expert
Attilio Renzulli	Forward translator 2	Translate the source document into the target language	Undergraduate student
Enrico Albertini	Back‐translator 1	Translate the common forward translation into the source language	Dentist Specialty in Orthodontics Lingual Orthodontics expert
Ovidiu Ionut Saracutu	Back‐translator 2	Translate the common forward translation into the source language	Undergraduate student
Debora Rossi	Expert panelist 1	Review the resulting translation, i.e., the (revised) common forward translation, focusing on semantic, idiomatic, experiential and conceptual equivalencies	Pedagogist Degree in Formative Sciences
Matteo Pollis	Expert panelists 2	Review the resulting translation, i.e., the (revised) common forward translation, focusing on semantic, idiomatic, experiential and conceptual equivalencies	Dentist PhD student Bruxism and Sleep‐Related Breathing Disorders expert
Marzia Segù	Expert panelists 3	Review the resulting translation, i.e., the (revised) common forward translation, focusing on semantic, idiomatic, experiential and conceptual equivalencies	Dentist Specialty in Orthodontics Bruxism and Sleep‐Related Breathing Disorders expert
Aurora Manfredini	Expert panelists 4	Review the resulting translation, i.e., the (revised) common forward translation, focusing on semantic, idiomatic, experiential and conceptual equivalencies	Undergraduate student
Chiara Bonanni	Expert panelists 5	Review the resulting translation, i.e., the (revised) common forward translation, focusing on semantic, idiomatic, experiential and conceptual equivalencies	Dental hygienist

As a first step, two forward translations of the source document from English into Italian were performed by two independent bilingual professionals. Based on consensus, a synthesis of the two forward translations was conducted by the first study coordinator and both forward translators. As a second step, two back translations of the common forward translation into English were performed by two independent, bilingual translators with and without specific medical/dental knowledge, respectively. A bruxism expert and both back‐translators fine‐tuned a common back‐translation by consensus. Finally, a bilingual language expert (i.e., the third study coordinator) compared both documents and identified the discrepancies of the common back‐translation against the source document.

### 2.2 Pilot Testing

The pilot on‐field testing on STAB comprehension and feasibility was performed at the School of Dentistry at the University of Siena, Siena, Italy, according to the procedures described in ‘Measurement in Medicine’ by De Vet et al., in order to assess the comprehensibility of the questions and response options, as well as the overall feasibility of the tool [14].

The target population included for pilot testing included:

- 20 patients (11 females, 9 males; age range = 18–69 years), 10 of whom were assessed as part of the clinical activities of the postgraduate master course in orofacial pain, and 10 of whom belonged to the general dentistry department. Details of the study population are shown in Table 2.
- 20 dental students (13 females, 7 males; age range = 18–27 years) who had received theoretical and clinical training on bruxism and orofacial pain within the last 2 years as part of the teaching activities of the School of Dentistry.
- 20 dentists (7 females, 13 males; age range = 26–60 years). Six of them were general practitioners, four specialists in orthodontics, three specialists in prosthodontists, three periodontists, three experts in orofacial pain and one specialist in oral surgery. All dentists attended either the Postgraduate Course in Orofacial Pain or the Postgraduate Course in Prosthodontic Technologies.

**TABLE 2:** Data of the study population (patients' characteristics).

Patient	Gender	Age	Level of school education	Dentistry department
Patient 1	Female	18	Tertiary education	General dentistry
Patient 2	Male	31	Postgraduate	General dentistry
Patient 3	Male	52	Tertiary education	General dentistry
Patient 4	Male	45	Postgraduate	General dentistry
Patient 5	Female	68	Tertiary education	General dentistry
Patient 6	Male	18	Tertiary education	General dentistry
Patient 7	Male	53	Postgraduate	General dentistry
Patient 8	Female	51	Tertiary education	General dentistry
Patient 9	Female	28	Postgraduate	General dentistry
Patient 10	Male	24	Tertiary education	General dentistry
Patient 11	Female	40	Postgraduate	Orofacial pain
Patient 12	Male	69	Secondary education	Orofacial pain
Patient 13	Female	42	Postgraduate	Orofacial pain
Patient 14	Female	19	Tertiary education	Orofacial pain
Patient 15	Male	54	Tertiary education	Orofacial pain
Patient 16	Male	35	Postgraduate	Orofacial pain
Patient 17	Female	37	Postgraduate	Orofacial pain
Patient 18	Female	65	Secondary education	Orofacial pain
Patient 19	Female	22	Tertiary education	Orofacial pain
Patient 20	Female	46	Tertiary education	Orofacial pain

Ethical approval was achieved by the Review Board of the Orofacial Pain Unit, Department of Medical Biotechnologies, School of Dentistry, University of Siena (#0024‐23). The derived Italian language version was used to that end, as produced following the above‐described rules for forward and back translation [12].

Any possible information that could be important to the improvement of the tool was collected by the examiners. To that end, the so‐called ‘probing’ method was used, where the patients as well as the participating dentists and dental students were questioned in detail by the examiners about the perceived content and interpretation of the items. A similar procedure was already used for the pilot testing of the original version of BruxScreen [15]. As such, the pilot testing will yield reports about the following main outcomes:

- Comprehensibility: All users were asked to report any limit to the good comprehension of the STAB, concerning the explanations as well as the questions and response options of the instrument. Any requests for additional explanation of terms were noticed to possibly better clarify the meaning.
- Feasibility: Participants were asked to report about the possibility to complete both the self‐report questionnaire and, for the dentists, the clinical assessment form of the STAB within a reasonable time span. Based on an arbitrary decision of the project coordinators, the time needed for completing the instrument was set at 20 and 15 min for the self‐reported and clinical part, respectively. In addition, the clinicians were asked to indicate if a tool like this would be easy to implement in the everyday dental setting.
- Miscellaneous: All participants to this pilot testing phase were asked to indicate possible adjustments they would do to the items and/or the answers. For instance, a common problem that might emerge based on previous questionnaire validation studies is how to manage the answers to an item when the patient does not know the answer. Also, possible suggestions about strategies to rate and grade the scores were asked.

Based on the above outcomes, decisions to further adjust the instrument could be taken.

## 3 Results

As specified above, a total of 60 subjects (i.e., 20 patients, 20 dental students and 20 dentists) took part in the pilot test.

Regarding the comprehensibility, dentists and dental students reported a good comprehension of the instrument, concerning both the explanations and the questions and response options. Three patients (Patients # 5, 12, 18) indicated that they would appreciate some additional explanations of some terms regarding the medical area (e.g., closed lock, mandible bracing).

Considering the feasibility, the participants reported that the self‐reported questionnaire could be completed within a reasonable time span, especially (based on three dentists' annotations) considering the importance of the topic and the comprehensive evaluation of bruxism issues. Only in the case of three patients (Patients # 5, 12, 15), the expected time of 20 min was exceeded.

As part of the clinical activities of the Dental School, dental students and dentists were split into groups of two individuals, each of whom evaluated the same patients on rotating turns. Everyone stated that, despite the high number of questions, filling out the clinical part is intuitive and potentially useful for implementing knowledge both in the clinical and research setting. The time taken by the dental students was slightly higher than that of the dentists (approximately 15% more), even if everyone reported to stay within the expected time. In addition, the time reportedly spent by orofacial pain experts was a bit lower than dentists from other disciplines (approximately 10% less).

Furthermore, some patients indicated that they would have preferred less response options and, if possible, a lower number of questions, perhaps unifying some items and/or response options.

Four dentists (i.e., two general practitioners, one orthodontist and one periodontist) and eight dental students asked for an indication of how the responses to the STAB would be translated into management strategies for bruxism and when this would become necessary. Such observations were actually not specifically pertinent to the Italian translation, so they could be taken into consideration for any future STAB refinement strategies.

## 4 Discussion

This manuscript aimed to present and describe the process of forward–back translation into Italian and of pilot testing of the Standardised Tool for the Assessment of Bruxism (STAB), an instrument that was developed to provide a multidimensional evaluation of bruxism status, comorbid conditions, aetiology and consequences.

The STAB was the result of several years of debates among the authors, which followed the publication of the 2018 consensus paper on bruxism definition. As such, in line with the consensus definition itself, the STAB is considered a work in progress, ready for on‐field testing of comprehensibility and feasibility. The STAB is intended to yield a valid assessment of the frequency of the different sleep and awake bruxism‐related jaw‐muscle behaviours, as well as of the most common potential clinical signs, risk and aetiological factors, clinical consequences and comorbid conditions [8].

Concerning the face validity of the STAB (i.e., the degree to which the tool looks as though it is an adequate reflection of the construct to be measured), it was assessed subjectively by collecting feedback on the instrument from all authors of this manuscript and who created the STAB [8, 11]. Indeed, in the absence of any standards regarding how to assess face validity, the outcomes could not be quantified [14].

Pilot testing of this Italian version has confirmed the good face validity of the text, as per the report of the dental professionals and dental students who took part in the study. In addition, it highlighted some further interesting issues that are partly in line with a recently published article [15].

As regards comprehensibility, only a minimal share of participants requested additional explanations regarding some medical terms. It is interesting to note that this occurred only in older subjects, aged between 60 and 69 years, who could have been less familiar than others with some medical terms. Indeed, even if the translation was conceived to be as clear as possible, the absence in the Italian language of an analogue of the English words ‘bracing’ or ‘thrusting’ made the use of elaborated wording needed. In general, the authors of this paper decided that the text of the Italian version is sufficiently clear.

The feasibility was also considered good. Indeed, although the self‐reported sections are very comprehensive due to the inclusion of multiple domains to address many aspects of the bruxism construct, participants indicated that it is possible to complete it within a reasonable time span, namely, < 20 min.

Concerning the clinical evaluation, both dental students and dentists agreed in stating that the evaluation is intuitive and complete as well as useful for implementing knowledge both in the clinical and research setting. The choice to prioritise pre‐existing instruments for inclusion in the STAB is likely to play a role to enhance feasibility.

Interestingly, the time reported by experts in orofacial pain was less than that of specialists in other disciplines, who in turn, however, took less time than students. This could easily be explained by the different background on the topic that characterises the different groups of participants: from the less experienced (i.e., dental students), to the more expert ones (i.e., specialists in orofacial pain). Further testing on the time needed in relation to the difficulty of assessing any specific patients is needed.

In addition, the pilot test suggested that some of the participants would prefer a lower number of questions. To overcome this request, it must be noticed that a Toolkit has been published as an appendix to the original STAB publication, including the full version of the instruments that are totally or partially embedded in the STAB. This allows selecting specific sections of the STAB, depending on the specific clinical and/or research needs of the users. As an important note, as a further step of this translation procedure, the non‐English mother tongue users are invited to check the availability of the specific instruments in their language. Regarding the request of some students and dentists to have an indication of how the responses to the STAB would translate into management strategies for bruxism and when this becomes necessary, it is important to underline that the adoption of decision‐making algorithms for the STAB is premature. An elaboration of screening strategies and identification of possible cut‐offs for treatment‐demanding findings was beyond the scope of this assessment and is a much needed demand for the near future. After the adoption of PSG‐based cut‐off criteria to diagnose SB has been shown poorly useful in the clinical setting, it must be remarked that sleep and awake bruxism are better assessed on a continuum spectrum [9]. With time, mining of the collected data would help defining the clinical impact of the instrument.

Within these premises, it must be remarked that actually none of the very minor points raised by the pilot testers regarded specifically the Italian version, which was unanimously appreciated. The translation into Italian can thus be considered a faithful and culturally adapted version of the original English instrument, thus being ready for use. This is an important step for the standardisation of data collection and cross‐country/‐culture comparison.

The methodology used in this study for the translation from English to Italian judiciously follows the methodology already described in the literature [12, 16]. In this scenario, it is important to underline that, as suggested by several studies, the methodological norms related to transcultural adaptation of research questionnaires should not be restricted only to translation but should also include the back‐translation, face validation, cultural adaptation and validity with the aim to avoid errors in the idiomatic, semantic, cultural and conceptual equivalences of the instrument [13]. The multiple translations that could be made in different languages and the use of a dedicated tool will allow an improvement of knowledge on bruxism as a whole and a better communication between the different communities of bruxism experts and general practitioners as well as a better management of bruxism in the clinical setting.

## 5 Conclusion

Based on the outcomes of the pilot testing in patients, dentists and dental students as well as of the face validity assessment, the Italian version of the STAB can be introduced in the clinical and research settings. The next steps provide that an assessment of reliability, validity, feasibility and responsiveness to change should be performed, as part of needed procedures also for the original English version.

## Conflicts of Interest

The authors declare no conflicts of interest.

### Peer Review

The peer review history for this article is available at https://www.webofscience.com/api/gateway/wos/peer‐review/10.1111/joor.13882.

## Data Availability

The data that support the findings of this study are available from the corresponding author upon reasonable request.
